# Kinks in Urine Examination

**Published:** 1897-05

**Authors:** 


### KINKS IN URINE EXAMINATIONS.

There are several kinks in the examination of urine which are very useful.

While the urine is in the specific gravity the jar insert a long pipette, open at the top. The urine will rise in it to the height of that in jar. Place your finger on the top, lift it out and hold it about level, placing the smaller end against the inside surface of a test tube in which about three-quarters of an inch of nitric acid has previously been poured. A little practice will enable you to float the urine on the top of the acid in the most gentle manner.

Nitric acid may be floated beneath the urine in the same gentle manner. If the test tube is placed upon the top of the lower sash of a window and at one end of it, the ring of albumin when present will be clear cut.

If the tube is held before a piece of black velvet, the coagulum will be brought out very distinctly.

When testing urine for albumin, if you will have two tubes of urine, one of which is allowed to remain in its natural state, the changes following the processes of testing may be appreciated by comparison. If you place a rubber nipple over one end of a pipette, into which a pin is thrust, or a minute slit has been cut, the contents of the pipette of nitric acid will flow out very gently, or squeeze the bulb and the same result will follow.—*Medical Examiner*.

